# Dispersal history of the golden jackal (*Canis aureus moreoticus* Geoffroy, 1835) in Europe and possible causes of its recent population explosion

**DOI:** 10.3897/BDJ.7.e34825

**Published:** 2019-05-09

**Authors:** Nikolai Spassov, Ilya Acosta-Pankov

**Affiliations:** 1 National Museum of Natural History, Sofia, Bulgaria National Museum of Natural History Sofia Bulgaria

**Keywords:** European jackal history, dispersal, ecological requirements, South-Eastern Europe

## Abstract

**Background:**

Data on the historical distribution of the golden jackal in Europe and its primary habitats are scarce. There are many new data on the population explosion and the rapid spread of the in Europe. However, the main factors for this expansion, the core population and its routes of dispersal, remain controversial or insufficiently studied.

**New information:**

This study provides a profound analysis of the history of the jackal’s (*Canis aureus moreoticus* Geoffroy, 1835) occurrence in Europe, the factors limiting or those triggering its expansion on the continent. The analysis shows that the timing of the species appearance in Europe still remains unclear. Historical data show that the species is a typical inhabitant of South-Eastern Europe, with some pulsations within its core area, as well as extensions to the north and west of it in favourable periods. Nowadays, the increase of the species range in Europe is the largest documented population explosion on the continent. We argue that this expansion originates from only three core populations, the Peri-Strandja area and the Dalmatian coast in the Balkans and the east parts of Western Transcaucasia in the Caucasus. This population explosion is largely due to a unique combination of factors of an anthropogenic nature.

## Introduction

Until recently, the golden jackal (*Canis aureus moreoticus* Geoffroy, 1835) was an exotic carnivore for Europe. During the first half of 20th century, the species had a restricted distribution in South-Eastern Europe. Its rapid westwards expansion on the continent generated great interest and has resulted in many publications on this issue in recent years (e.g. Arnold et al. 2011, Šálek et al. 2013, Galov et al. 2015, Lanszki et al. 2015, Penezić and Ćirović 2015, Rutkowski et al. 2015, Trouwborst et al. 2015, Ćirović et al. 2016, Comazzi et al. 2016, Musabekov et al. 2016, Krofel et al. 2017, Newsome et al. 2017, Markov et al. 2018). Yet, it remains a poorly studied species. For example, the time of its appearance on the continent, despite several statements, remains controversial (Jullien 1968, Tranier 1973, Hosey 1982, Spassov 1989, Demeter and Spassov 1993, Sommer and Benecke 2005, Lapini et al. 2011).

## Materials and methods

A total of about 700 *Canis* remains (stored in the National Museum of Natural History, Sofia) were examined and identified (by NS) in the search for jackal characters amongst them. They originate from 29 South and North Bulgarian archaeological sites from the Neolithic (8000 years BP) till the Medieval Age. The detailed analysis of literature on the history of the presence of the species by year allowed us to restore the species distribution area from the late 19^th^ century to recent times, its pulsation over time and the establishment of the core populations, from where the modern expansion of the species on the continent began. Additionally, we used the published data on genetic structure and variability of the European jackal.

**Figure 1.**
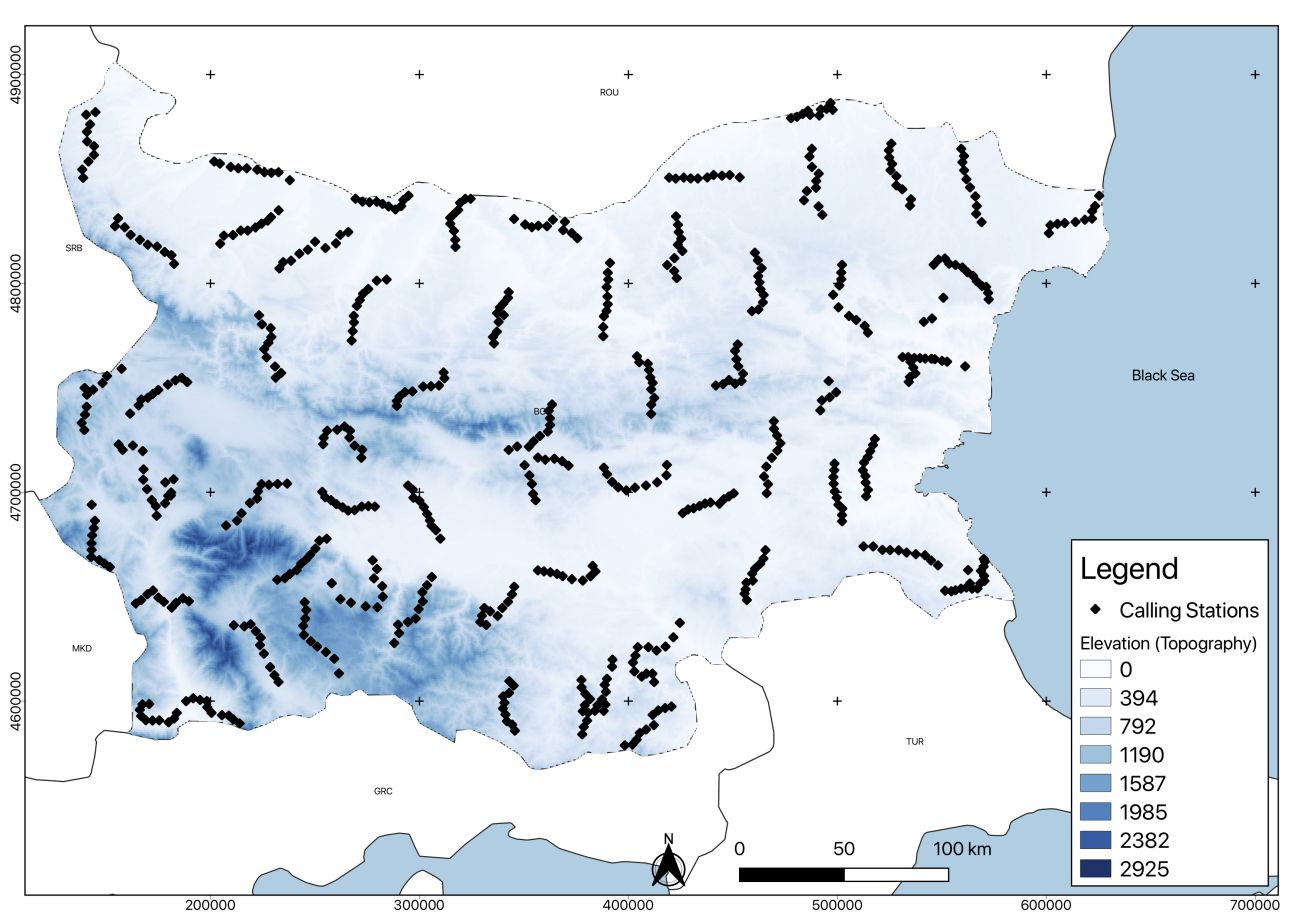
Geographic location of the calling stations (CS) during the bioacoustic survey. This map was created with QGIS 3.4.4 (QGIS Development Team, 2019).

Experimental fieldwork data, collected between 2014 and 2017 in Bulgaria, complemented the analysis of the factors limiting the spread of jackals’ populations in the country, as well as those which might be responsible for its expansion. The analysis of this case study was used as an explanation for the responsible factors for the whole continent. We collected data on jackal presence, aiming to relate the habitat characteristics variation with the positive answer proportion. To determine presence, we used a play-back bioacoustic method (Giannatos et al. 2005). We selected 621 calling stations (CS), located across Bulgaria (Fig. 1) for broadcasting, using a pre-recorded jackal howl with a 60 Watts power megaphone, which included USB, SD inputs and MP3 player software. The calling stations were organised in transects of 10 points, separated by at least 3 km from each other. The criteria used for the location of the CS was the last jackal density population reported in literature for Bulgaria (Spassov 2007). Based on this and the accessibility of the terrain, we selected a representative sample for each area, 189 CS (in high population density area), 271 CS (in medium population density area) and 161 CS (areas with rare presence or even absence). The vocalisation type used in the recording for stimulation was a long-distance call (Kolar et al. 2005), with two jackals. It was recorded in June 2014 near the town of Chernevo in North-Eastern Bulgaria (Varna Region) and had a duration of 30 seconds. The same record was used in all the calling stations. Every broadcast howl was followed by a 3-minute pause and repeated five times for each calling station. With a compass, we determined the direction for each positive answer and estimated roughly the distance as close (0-500 m), medium (500-1000 m) or far (1000-1500 m). The compass data, indicating the direction for each answer and the estimated distances in the field (close, medium and far), were used for mapping the point where the response came in each calling station using the azimuth and distance plug-in of a GIS software QGIS 3.4.4 (QGIS Development Team 2019); the total survey area (4389 km^2^) was also calculated with the same software. An exploratory analysis was made, relating the positives answers and sample proportion with different habitat factors (univariate descriptive analysis), such as the water source distance, altitude (topography), vegetation, distance to closest village, anthropogenic influence and wolf distribution.

## Taxon treatments

### Canis aureus moreoticus

(Geoffroy, 1835)

#### Dispersal history of the golden jackal in Europe

**Earliest data on jackal presence on the continent.** Data on the historical distribution of the golden jackal in Europe and its primary habitats are scarce. Despite the many new data on the population explosion and the rapid spread of the species across Europe, the main factors for such population expansion remain controversial. The core population/local populations and the routes of dispersal remain insufficiently studied.

Hosey (1982) suggested that the jackal could have reached Europe from the east at the end of the Pleistocene, a hypothesis that appears justified from the zoogeographic viewpoint. Theoretically, the jackal could penetrate in Eastern Europe in two ways, that correspond to the potential paths at the end of Pleistocene and Holocene: along the northern Black Sea coast and through the Bosporus (Spassov 1989). The opening of the Bosporus took place 7600 cal BP or, more probably, 9300 cal BP (Yanchilina et al. 2017).

The area of distribution of the European subspecies *Canis aureus moreoticus* (Geoffroy, 1835) during the first half of 20^th^ century occupies a relatively vast territory from the Balkans, which is the initial European territory, up to Anatolia and Caucasus. There is no significant difference in the colouration pattern and other features across the various subpopulations living in this area (Pocock 1938, Heptner et al. 1967, Demeter and Spassov 1993). Despite several old and relatively recent statements about Late Pleistocene presence of the golden jackal in Europe, there are no fossil records of *Canis aureus* found on the continent (Demeter and Spassov 1993, Lapini et al. 2011). The Pleistocene climate may have been inappropriate for this species (see below) and there are no data on fossil records of *C. aureus* in the proximity of the eastern and south-east European territories between the end of the Pleistocene and the beginning of the Holocene: according to Vereshchagin (1959) and Baryshnikov (1986), there are no confirmed remains of *C. aureus* from the Pleistocene of the Caucasus and Transcaucasian Region and that the species possibly reached this area rather recently, in the Middle Holocene. Several doubts, regarding the presence of the jackals in the Holocene of Europe, have been expressed. These concerns, however, were based on problematic remains and turned out to be erroneous (see Spassov 1989). The available remains reported as “subfossil jackals” from Bulgaria were also revised years ago. They clearly belong to dogs (Spassov 1989). More recently, this question was raised again by Sommer and Benecke (2005), who cited Jullien (1968) and Tranier (1973) reporting that the species was found in the Neolithic in Greece. These claims were based, again, on unsure, scarce bone remains which have not been described, compared nor figured. In the last decades, one of the authors (N. S.) observed hundreds of skull remains of *Canis* from many Neolithic to Medieval sites in Bulgaria (see Material and methods). No jackals were determined there. All of this suggests that, until now, there is no proof about discovered fossil or subfossil remains of jackals in Europe and that the claim (Sommer and Benecke 2005) about the species presence in the Holocene of Greece (widely cited, e.g. Zachos et al. 2009, Rutkowski et al. 2015, Trouwborst et al. 2015, Krofel et al. 2017, Lanszki et al. 2018) is more than doubtful, as it is not based on surely determined remains.

It possibly penetrated in the Early Holocene and lived as a rear animal without economic value for humans and has almost never been hunted (that is why it has not been found yet); as a good swimmer, it could penetrate from the east even in historical times (Spassov 1989). It could have been introduced most probably in post-antiquity as a pet animal (Keller 1909). For the moment, in the absence of evidence, this appears to be a possible hypothesis.

The first record of a jackal in Europe (the south-eastern and eastern parts of the continent) dates back to the Middle Ages. The earliest reliable historical data are from the end of the 14^th^century (the vicinity of Sofia), from Turkish chronicles, during the siege of the town (Gueorguiev 1983). There is an anecdotal story about Venetian sailors who introduced jackals in the 15^th^century on the Dalmatian coast from North Africa (Kühn 1935, Milenković 1987). This story which, as it seems, has caused diplomatic complications at that time, finds confirmation in an official letter between the leaders of Zara\Zadar and Venice, which is stored in the national archives in the Zadar City (Miklós Heltai in lit.). The North African origin is not supported by taxonomic studies (Kryštufek and Tvrtkovic 1990), but it is interesting to note that the Dalmatian local population is more distant morphologically and genetically from the other Balkan ones (Kryštufek and Tvrtkovic 1990, Fabbri et al. 2014). For Ukraine, historical data exist from the so-called Cossack Era (16^th^-18^th^century) (Zagorodniuk 2014).

**Figure 2.**
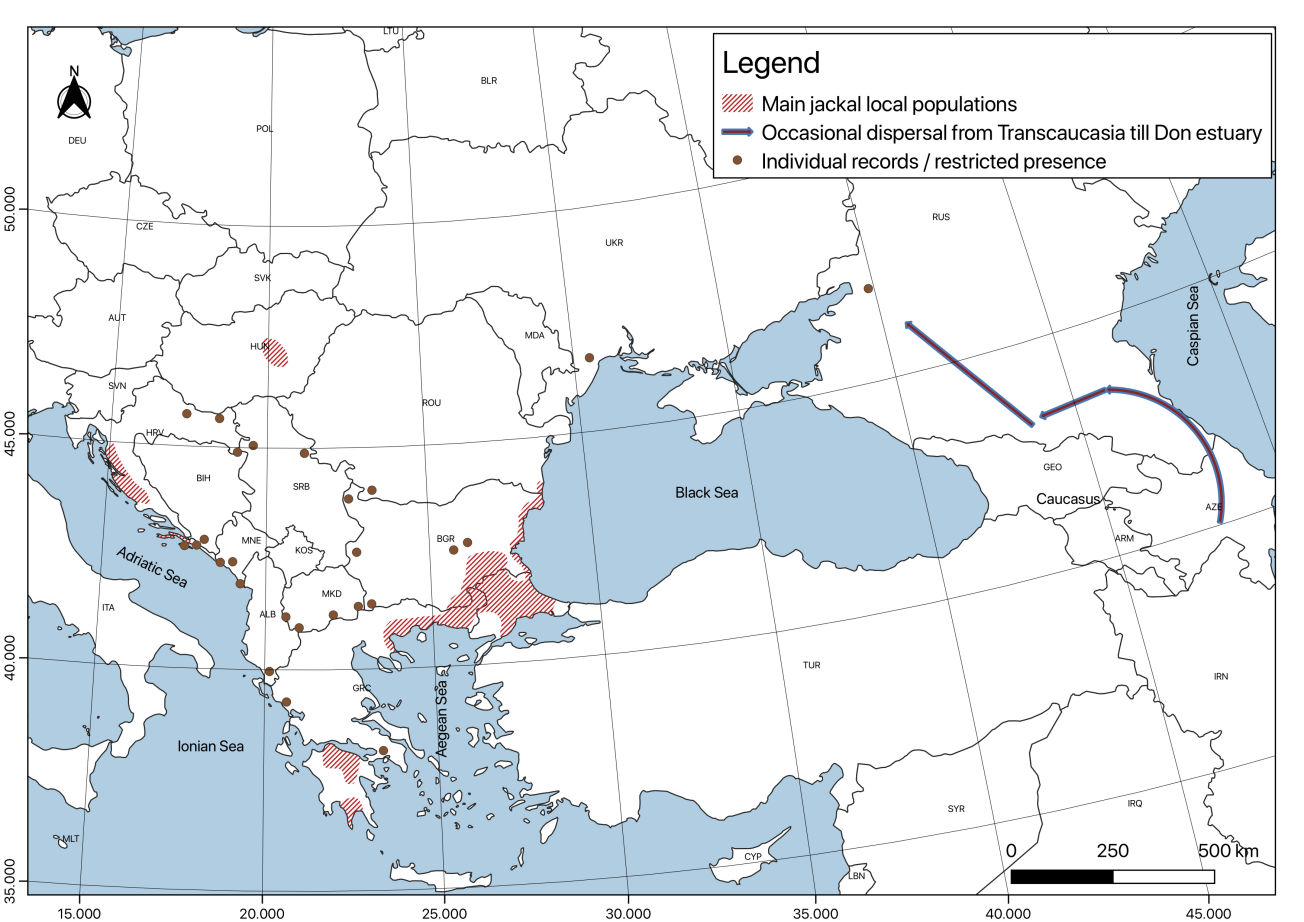
Approximate distribution of the golden jackal in Europe from the end of the 19^th^ until the 1930s of the 20^th^century. Based on data from Calinescu (1930), Ehik (1938), Atanassov (1953), Vasiliu (1961), Pomakov (1981), Milenković (1987), Demeter (1984), Spassov (1989), Demeter and Spassov (1993), Kryštufek et al. (1997), Musabekov et al. (2016). This map was created with QGIS 3.4.4 (QGIS Development Team 2019).

**History of jackal distribution from the end of the 19^th^ until the 1930s of the 20^th^ century (Fig. 2).** In Europe, the jackal was mainly distributed within the Balkans (Blasius 1857, Atanassov 1953, Pomakov 1981, Milenković 1987, Spassov 1989, Demeter and Spassov 1993, Kryštufek et al. 1997, Giannatos et al. 2005). The more stable local populations were presented in the Thracian Region (Northern Thrace in Bulgaria, Eastern Aegean Thrace in Greece and Western Thrace in European Turkey), Dalmatia and Peloponnesus, with pulsations/expansions to the west/northwest during favourable periods: it existed on the Bulgarian Black sea coast and occasionally reached West & North Bulgaria and possibly even North Serbia (Atanassov 1953). The population occurred mainly on the southern coast, covering the territory between the Marmara and Aegean Seas (from Istanbul to Chalkidiki, interrupted along the western Greek coast and Peloponnese); to the north, the distribution extended between the Eastern Rhodope Mts., the Sakar hills and the Strandja foothills from the Bulgarian and Turkish Territories, as it continued northeast along the Black Sea coast of Bulgaria to Varna and even to the Romanian border; to the west through mountainous border territories from South-Western Bulgaria to the foothills between today's Republic of North Macedonia and northern Greece.

On the Adriatic coast, there were fragmented small subpopulations (apparently with temporary connections between them): from the Greek coast south of Ioannina to small spots along the Albanian coast and, from there, to the Dalmatian coastal area (including the Pelješac Peninsula and Korčula Island).

In Romania, the jackal was also an occasional visitor from Bulgaria, crossing the frozen Danube River during severe winters (Vasiliu 1961, Vasiliu and Şova 1968). It was reported for the first time in this period in 1929 in Wallachia, in front of the town of Lom (in North-Western Bulgaria), but in the 1920s, it also appeared in Romanian Dobrudja (Calinescu 1930, Atanassov 1953). A small localised population existed in Hungary at the beginning of the 20^th^ century (Ehik 1938, Demeter 1984). From there, it seemed to have disappeared in the early 1940s.

In Eastern Europe, the species occasionally penetrated from the Caucasus to the Don River estuary (Musabekov et al. 2016).

There are two very different tendencies of the population dynamics which are treated in this article as two periods: from the middle of the 20^th^ century till the 1980s, a great reduction in the population was observed. It was followed by the beginning of a population expansion.

**Figure 3.**
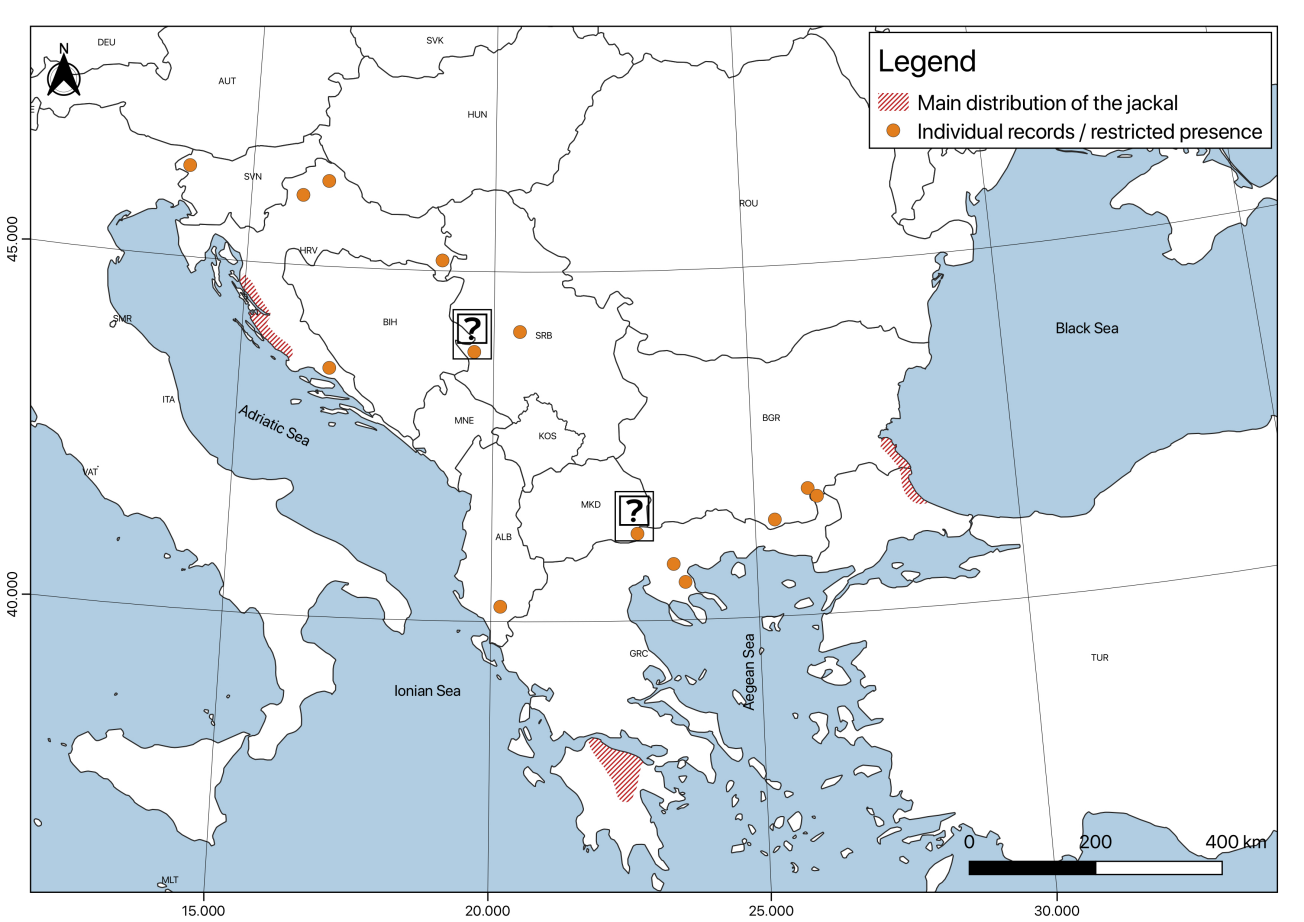
Approximate distribution of the golden jackal in Europe from the second half of the 1950s until the beginning of 1960s (the population minimum: see the text). This map was created with QGIS 3.4.4 (QGIS Development Team 2019).

**History of jackal distribution from the second half of the 1950s until the beginning of 1960s: population minimum (Fig. 3)**. The jackal population shrunk, the peripheral small populations gradually disappearing from Hungary (unconfirmed individual records from 50s: Tóth et al. 2009), Romania, Republic of North Macedonia, Serbia (except for some small isolated spots) and Bosnia. In fact, in Bulgaria, the reduction of the species numbers was noticed long before this (Atanassov 1953, Milenković 1987, Demeter and Spassov 1993, Kryštufek et al. 1997). In the 1950s, jackals were reported in the southern part of Romania and in north-eastern Romania near Piatra Neamt and Focsani (Banea et al. 2012). These cases were probably related to occasional dispersals from Bulgaria. It disappeared in many areas during the 1960s because of habitat loss and poisoned bait (Spassov 1989). It remained in localised subpopulations within the Balkans, which we can call basal (core) populations. From these population nuclei, in the late 1960s and most notably in the 1970s, stabilisation and expansion to the north and the west began to occur.

The core populations could be defined as follows: 1. Strandja coastal area of Bulgaria and Turkey (probably also some areas in E. Rhodope-Sakar Mts.) (Pomakov 1981, Spassov 1989); 2. Fragmented Adriatic population, mainly in Dalmatia (in Slovenia from the early 1950s, most likely entering from Croatia, however, disappearing quickly afterwards) (Demeter and Spassov 1993, Milenković 1987, Kryštufek et al. 1997); 3. Strimon-Chalkidiki Region (skeletal population, possibly including vagrants around Dojran lake); 4. Peloponnese population (isolated no later than the end of the 19^th^ century).

Most important in relation to the further expansion of the species are the first two core populations. The astonishingly high current number, over 30,000 individuals (Stoyanov 2013) and high-density areas, 5.66 to 7.08 territorial groups per 10 km^2^ (Acosta-Pankov et al. 2018), represent additional signs that the territory of Bulgaria is related to the core area of the population dispersal in Europe.

**Figure 4.**
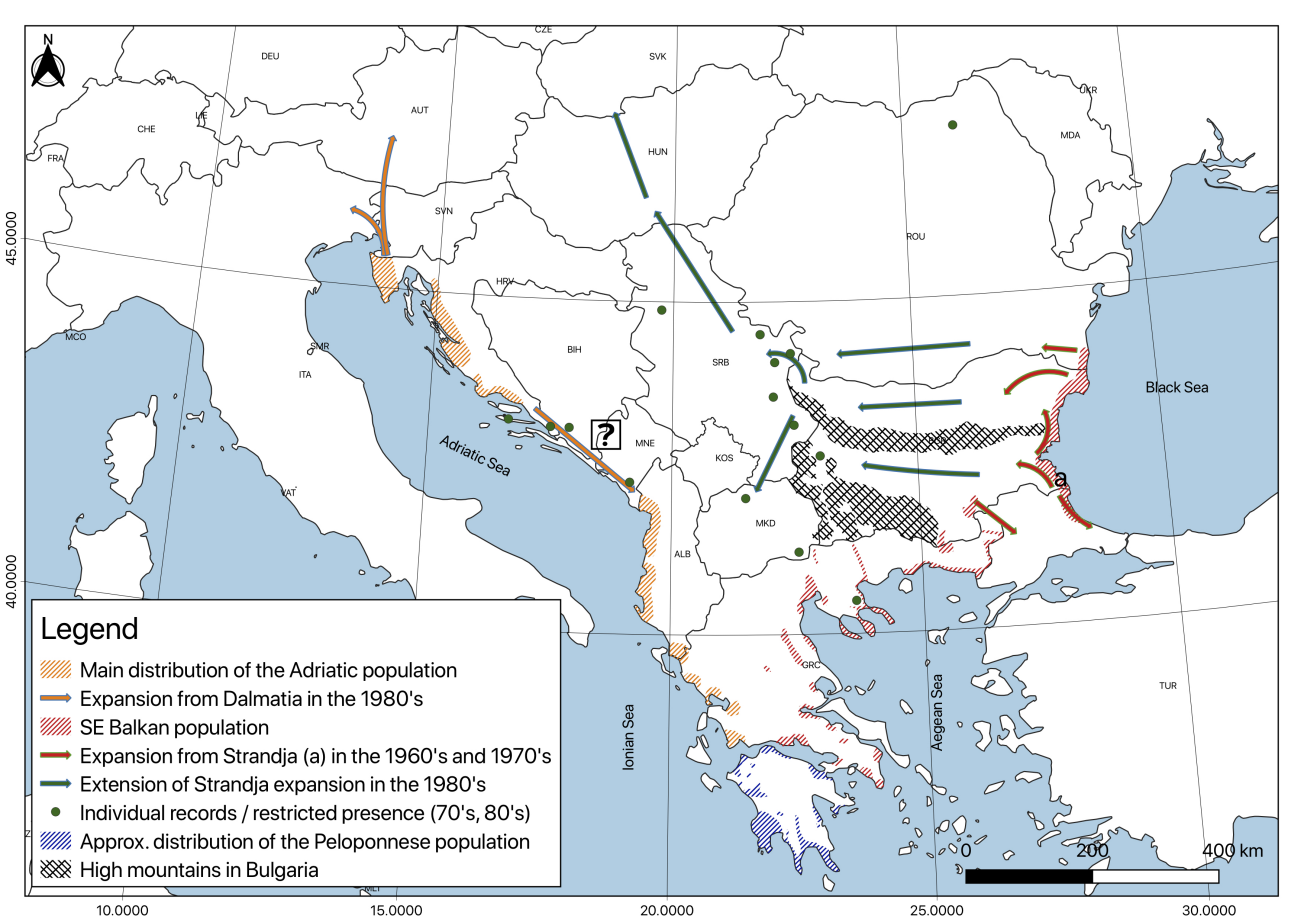
Approximate distribution of the golden jackal in Europe at the beginning of the expansion: population explosion of the Strandja and the Adriatic core populations. This map was created with QGIS 3.4.4 (QGIS Development Team 2019).

**Beginning of the expansion on the continent: population explosion of the Strandja and the Adriatic core populations (Fig. 4).** Our distribution analysis showed that the most powerful expansion of the jackal began from the Strandja core population and is continuing to the present time.

Bulgaria: In the late 1960s and early 1970s, after poison bait was banned and the protection of the species occurred (in 1962), the expansion began to the north (along the Bulgarian Black Sea coast) and to the west (in the Thracian lowlands and to the west of south Dobrudja) avoiding the high mountains (Pomakov 1981, Spassov 1989, Demeter and Spassov 1993). From the Strandja-Sakar region, the expansion increased possibly also to the south, influencing the distribution of the population in Eastern Thrace, Turkey (there is no reliable data for this period) and Western Thrace (judging from the map of distribution of the species in Greece: Giannatos et al. 2005). In the first half of the 1980s (some data indicate individual vagrants before this time: Sofia plain), the jackal reached Western Bulgaria excluding the border mountainous territories and south-western parts, where large mountain massifs occur (Genov and Wassilev 1989).

Romania: In the early 1970s, the jackal reached Romanian Dobrudja again (Kryštufek et al. 1997). In 1970, footprints were observed in Romania at the Humor Monastery’s hunting terrain and two jackals were hunted near Voronet (Bucovina); between 1971 and 1975, jackals were registered in the Buzau County in Dedulesti and Stefanesti near Bucharest (Angelescu 2004).

Serbia, Hungary and Slovakia: The species apparently spread westwards, reaching these countries through the lower Danube River plain, coming mainly from Bulgaria, but also from Romania. In the 1980s, the Romanian population expanded to the west and north, from where it reached again, in the early 1980s, Serbia (some individual records exist from the late 70s: Milenković 1987) and Hungary (Demeter 1984, Kryštufek et al. 1997, Tóth et al. 2009). It reached Slovakia in 1989 (Arnold et al. 2011). Markov et al. (2018) found a low epigenetic diversity of the jackal populations from Bulgaria and Serbia to Hungary. This indicates that the long-distance expansion from Bulgaria to Hungary is very recent and has started from a small population within a limited region.

Republic of North Macedonia: In 1989, the species was registered in the north-western part of the country (Kryštufek and Petkovski 1990), apparently coming from Serbia. Entering from Bulgaria was a much more difficult occurrence because of the low population density of jackals (until today) in South-Eastern Bulgaria owing to the unfavourable conditions in the border mountain areas.

The combination of several factors (Spassov 1989) could explain the explosion of the Bulgarian population after the 1960s, which was especially important for the further dispersal of the species in Europe: 1. The prohibition of poisoned bait and the temporary protection of the species in 1962; 2. In the 1970/80s, the hunting/farming in Bulgaria was amongst the best in Europe; fallow deer and roe deer fawns and wild game carcasses represented abundant additional food; 3. The well-developed free sheep-breeding (dead animals represent additional food); 4. The intensive plantations of pine forests in unfavourable areas where they cannot develop: creation of a widespread mosaic of impassable shrubs (shelters); 5. The wolf was still missing in the 1970s and the first half of the 1980s from the territories invaded at this time by the jackal (see: Spiridonov and Spassov 1985).

In the 1980s, probably from the Dalmatian core population (Kryštufek and Tvrtkovic 1990, Kryštufek et al. 1997), some individuals reached Northern Italy (1985) (Lapini and Perco 1988), Slovenia (1985) (Kryštufek et al. 1997) and Austria (1988) (Hoi-Leitner and Kraus 1989). Judging from the growth of the population on the Dalmatian Adriatic coast, the expansion probably reached Albania (see the map of the distribution in Kryštufek et al. 1997), where the status of the species is still cryptic and the population is not abundant (Giannatos 2004, Arnold et al. 2011). Genetic analysis confirms that the Italian population originates from Dalmatia and from Slavonia simultaneously (Fabbri et al. 2014), where the population comes from Bulgaria, via Romania and Serbia (Banea et al. 2012). The Austrian population (possibly also the Slovenian one) may have mixed origins (from the Dalmatian, but also from the Strandja core population, through Serbia): an Austrian vagrant jackal is genetically indistinguishable from the Serbian animals regarding both mtDNA and microsatellites (Kusza et al. 2018). This is interesting, because it has been assumed that the jackals found in Italy, Slovenia and Austria originate from the Istria Peninsula and North-Western Croatia (Kryštufek et al. 1997).

**Figure 5.**
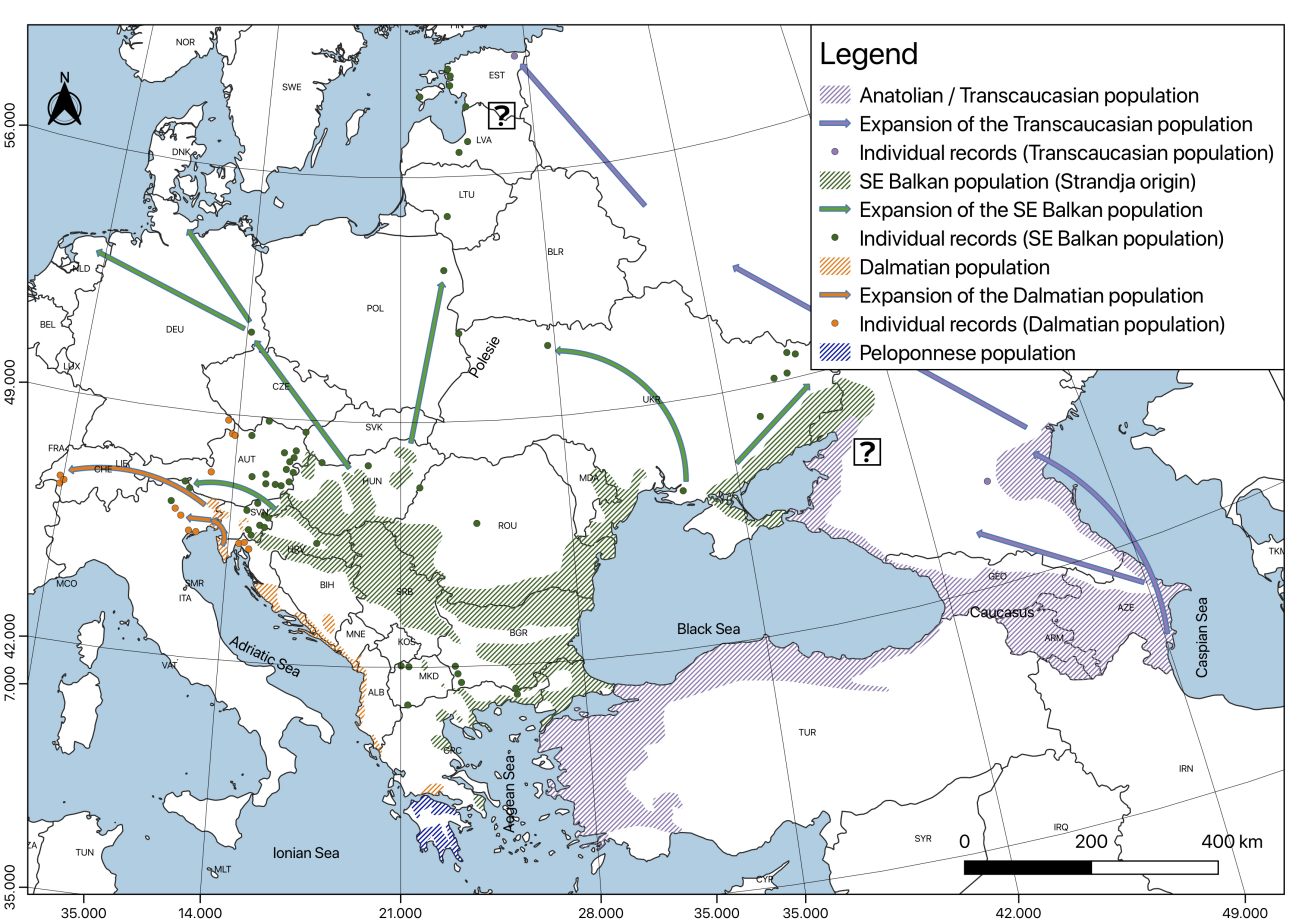
Approximate distribution of the golden jackal in Europe from the end of the 20^th^ until the beginning of the 21^th^ century: continuance of the expansion. The basic contour of the distributions of the species (individual records incl.) are from Trouwborst et al. (2015), Ambarli et al. (2016), Krofel et al. (2017) and Giannatos et al. (2018) with modifications and additions. This map was created with QGIS 3.4.4 (QGIS Development Team 2019).

**Continuance of the expansion from the end of the 20^th^ until the beginning of the 21^th^ century: (Fig. 5).** In this last period, there are dense populations of the jackal throughout the main territory of Bulgaria and Serbia, practically all the Wallachian Plain in Romania and northwards and westwards to regions of Europe where it has never occurred naturally, such as Germany (1996) and the Czech Republic (2006) (Arnold et al. 2011). Vagrant specimens have extended to Switzerland and the Baltic Region and it was reported for Estonia in 2013, the Netherlands (2015) and Denmark (2016) (see: Pyšková et al. 2016). As noted above, the population genetic research shows that the jackals from Italy, Slovenia and Austria have mixed origins from Dalmatia and Slavonia (Kusza et al. 2018) (in Slavonia, jackals likely have SE Balkan origin). From Romania, the species reaches Ukraine: the first record has been reported in 1998 for the Danube River Delta (Odessa Region). From there, the most powerful wave of dispersal was directed to the north, in the Polesie Region and recently from this region, most probably the jackal has reached Poland, Belarus, Lithuania and Estonia at the beginning of this century. In the first decade of our century, the jackals, originating from SE Europe, have reached not only Western Europe but have migrated to the east, reaching the border with Russia (Zagorodniuk 2014). The Transcaucasian population appears to be expanding similarly in the late 20^th^ century, reaching, at the 20^th^/21^st^ century, the eastern parts of the North Caucasus and the Saratov Region, also entering Russia from there. At the beginning of the 21^st^ century, this population has reached to the west the Ciscaucasia regions of Stavropol and Krasnodar in Russia (Musabekov et al. 2016). Thus, in recent times, the European population, expanding in the late 1960s from the Strandja core population, has made contact with the Caucasian (Trans-Caucasian) population of *C. a. moreoticus*, at the border between Ukraine and Russia. The genetic structure of the studied Lithuanian sample suggests that part of the Baltic jackals originate, as could be expected, from the population from South-Eastern Europe, while others (from the Estonian sample) originate from the Caucasus Region (Rutkowski et al. 2015), supporting the statement about a Caucasian (Transcaucasian) expansion in recent times.

## Discussion

**Factors limiting the spread of golden jackal population.** The presence of the species is related to certain ecological requirements, to which *C. aureus* has adapted during its evolution. Amongst the main factors limiting the occurrence of the golden jackal are (Spassov 1989):

1. Natural ones: deep snow, extreme frosts, large forest massifs, heavily intersected (steep) relief and the presence of wolves. This last factor was discussed largely in the works of Genov and Wassilev (1989), Kryštufek and Tvrtkovic (1990), Giannatos et al. (2005). According to Krofel et al. (2017) and Newsome et al. (2017), wolf extermination could be the key factor that could enable the expansion of jackals throughout Europe. We can agree that the wolf has an undoubted role as a limiting factor. However, this factor is hardly the only one responsible for the elimination of jackals from certain territories. The habitat of the jackal overlaps with that of the wolf on large territories in South Asia. Top predators and meso-predators exist in the same habitats on vast territories (Africa), when food and hiding places are sufficiently abundant. In the Eastern Rhodopes (Bulgaria), there is evidence about the co-existence of wolves and jackals feeding from the same carcass (Kurtev 2016) and personal data from a bioacoustic survey in Bulgaria (see also below), wherein at one calling station, we have simultaneous answers from jackals and one wolf, at a distance not more than 500 m from each other. In this region, the climate is relatively soft, the mountains are not high and the forest and open areas create a mosaic landscape, so that both species in the area are in large numbers. In cases where the jackal avoids wolf areas, it should be noted that this is related not only to the role of the wolf as a mega-predator but also the fact that the wolf currently inhabits wooded and inaccessible mountainous areas that are not suitable as habitats for jackals. A combination of the first four major environmental factors, mentioned above, exists in these mountain areas. The jackal has relatively short legs and its paws have a fairly small surface area, so it is not well suited for deep snow, while, at the same time, its fur is not adapted for heavy winters (Aliev 1968, Taryannikov 1974, Vereshchagin 1959, Heptner et al. 1967). Due tothe deep winter snows, extreme frosts, large forest massifs, intersected (steep) relief, mountain habitats are not suitable for the species (Spassov 1989, Demeter and Spassov 1993). An exception is the *C. a. moreoticus* habitat in the Talysh Mts. (Azerbaijan) where jackals’ tracks have been observed in a dense forest at 800 m, occasionally reaching 1800 m a.s.l., likely owing to the subtropical climate influence (Aliev 1968).

2. Anthropogenic factors (strongly affecting the species existence in the first half of the 20^th^ century): destruction of the habitats (scrublands and reeds) and direct destruction, mainly by poisoned bait (Spassov 1989).

**Bioacoustic monitoring results as a test of the indicated natural factors.** We recorded 328 positive jackal answers at 621 calling stations in all Bulgaria (see Materials and methods). Based on the positive jackals' answer proportion, related to the sample proportion, we analysed the factors limiting the spread of jackals’ populations in Bulgaria. The role of the human population and activities as a factor will be considered (see below) about the factors related to the jackal expansion. The analysis of these results for the country can be used as a case study to explain the responsible factors for the dynamics of the population on the entire continent.

**Figure 6.**
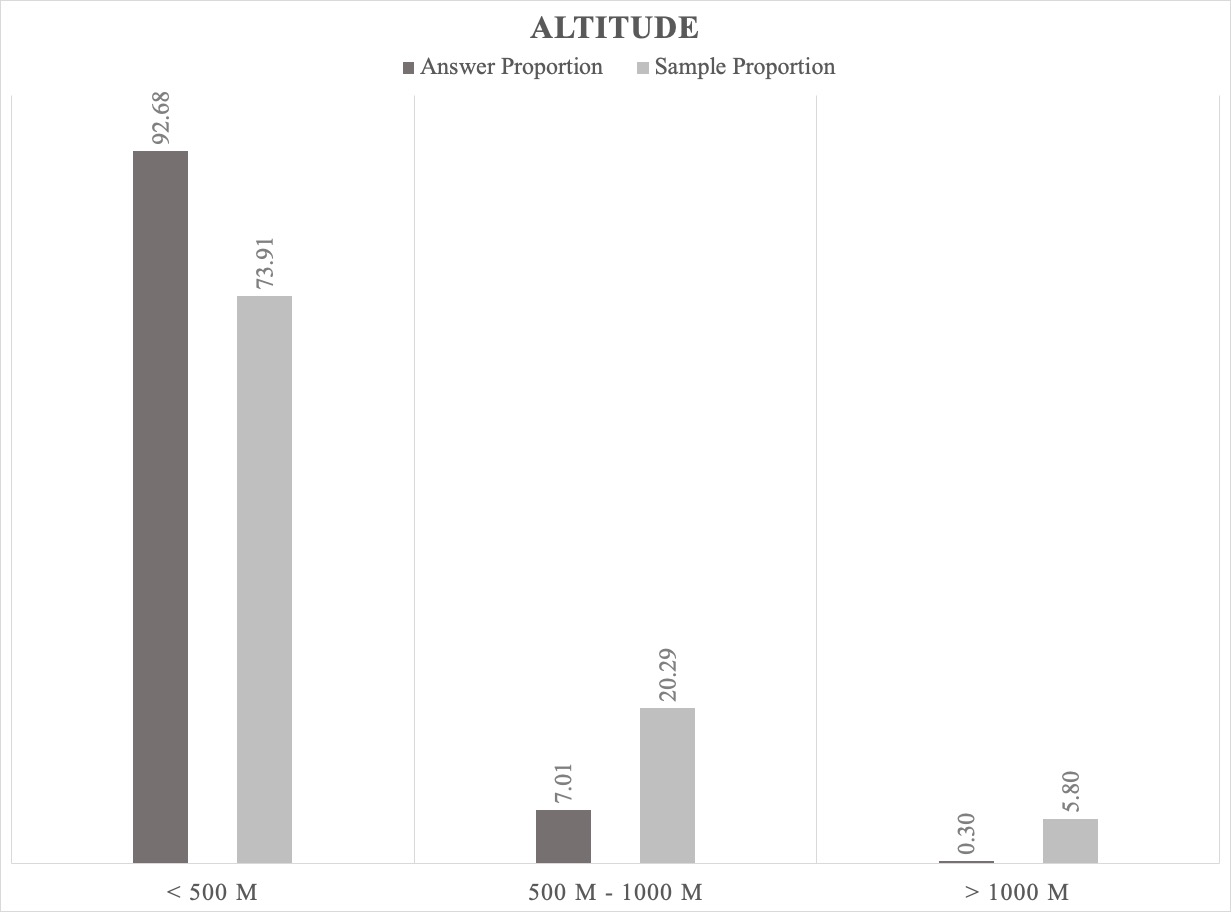
Proportion relationship between the jackals' positive answers and sample altitude (topography) as a factor that influences the jackal distribution.

**Figure 7.**
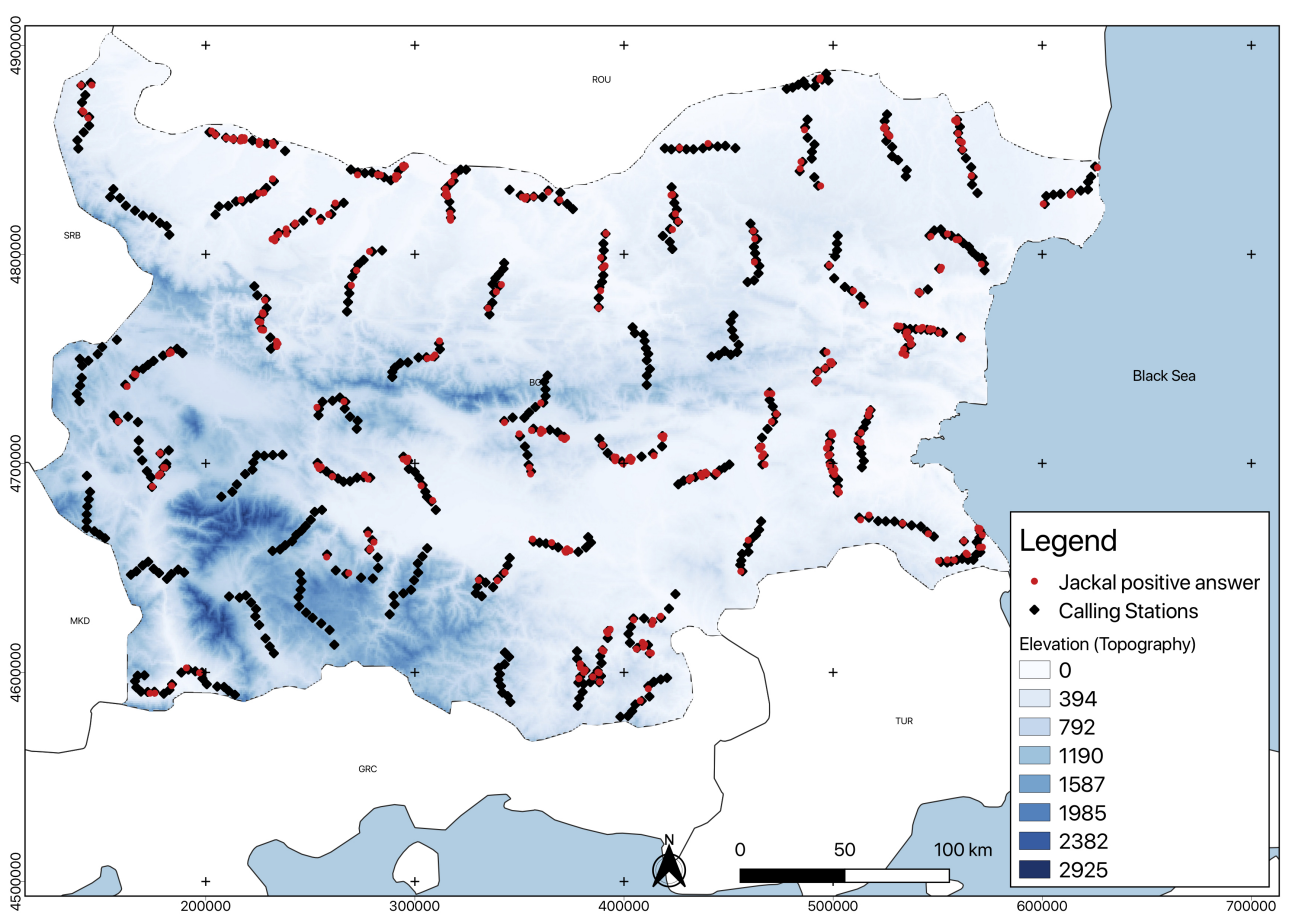
Location of the jackals’ positive responses in relation to Bulgarian topography. This map was created with QGIS 3.4.4 (QGIS Development Team 2019).

**Figure 8.**
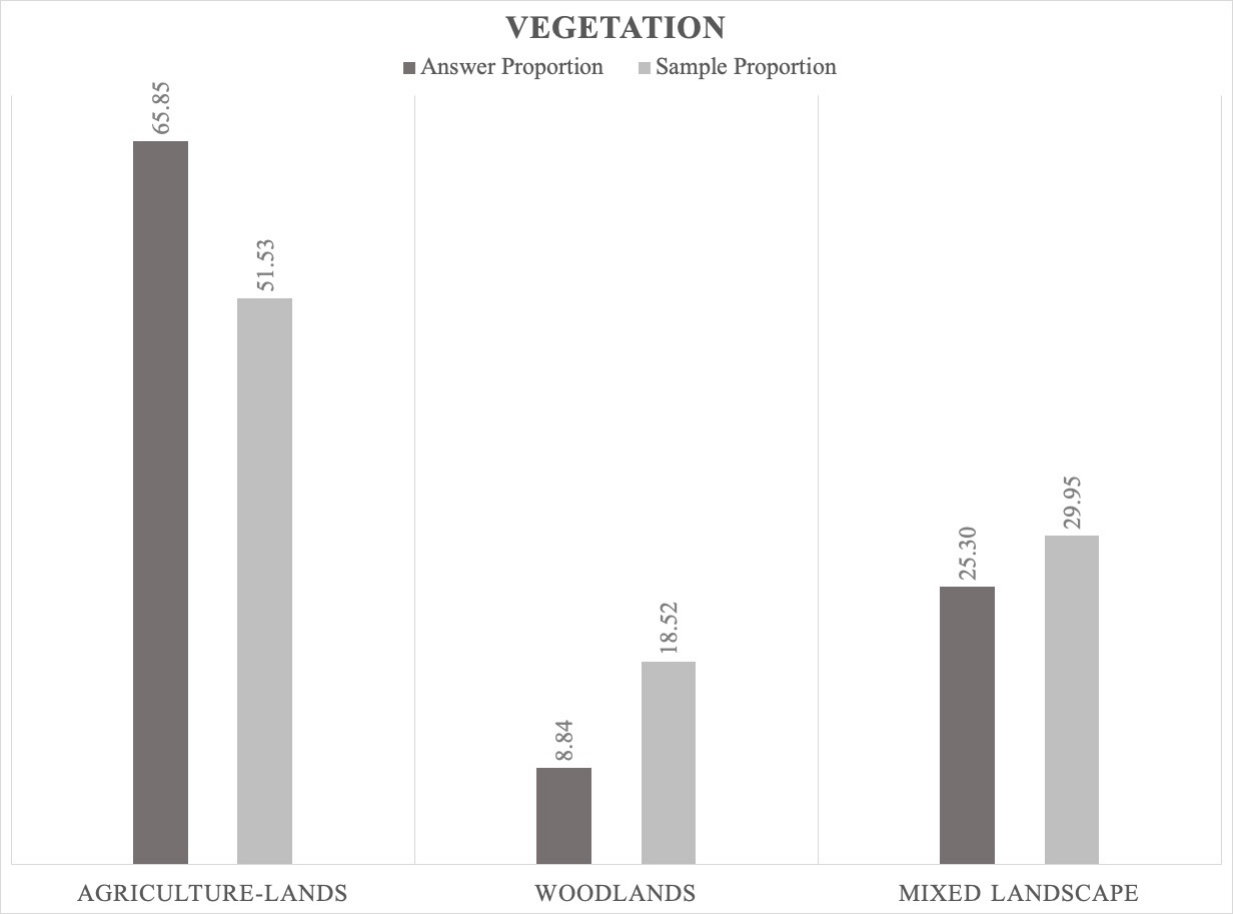
Proportion relationship between the jackals' positive answers and sample vegetation type as a factor limiting the jackal distribution.

**Figure 9.**
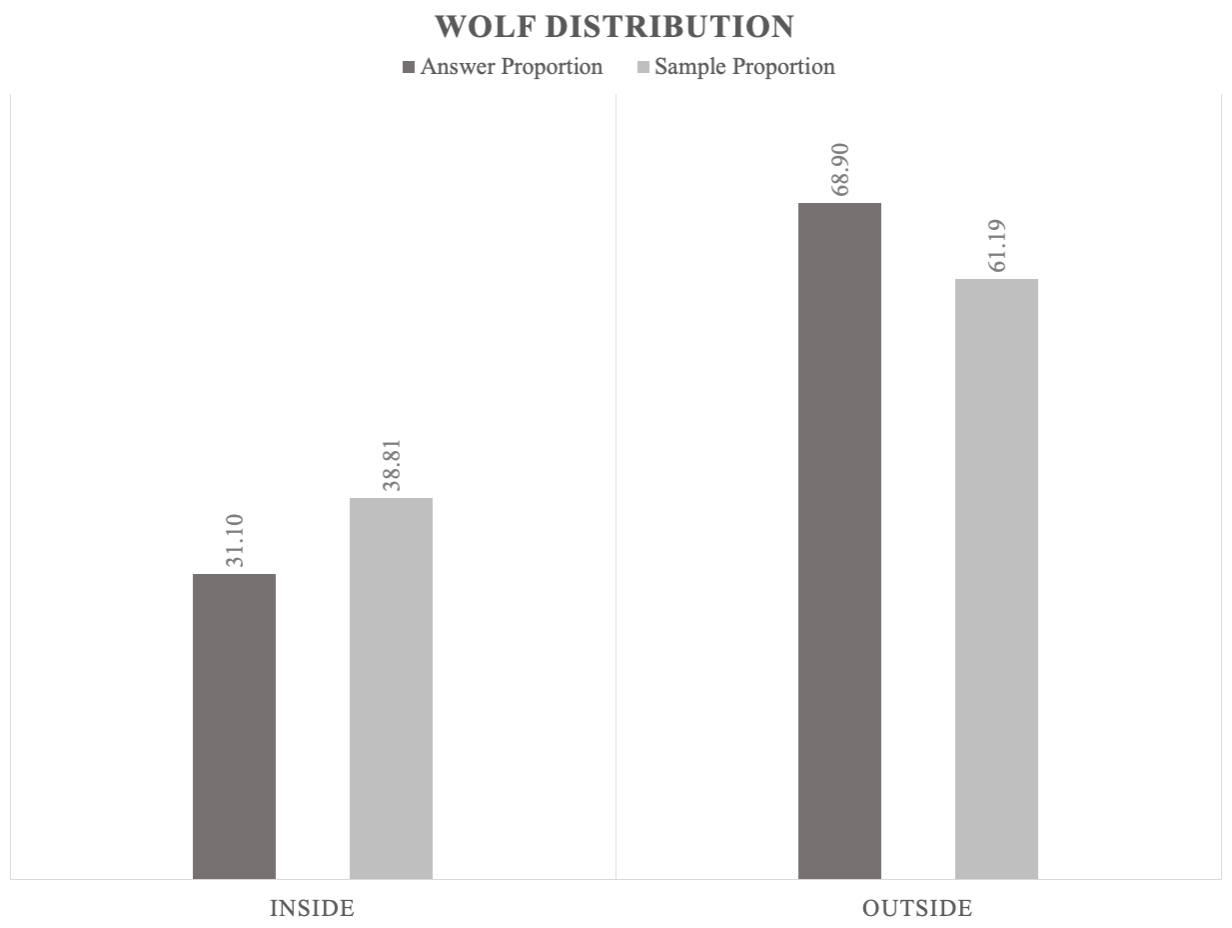
Proportion relationship between the jackals' positive answer and the sample regarding the wolf distribution (inside/outside wolf reproductive territory Spiridonov and Spassov 2015) as a factor that influences the jackal distribution.

**Figure 10.**
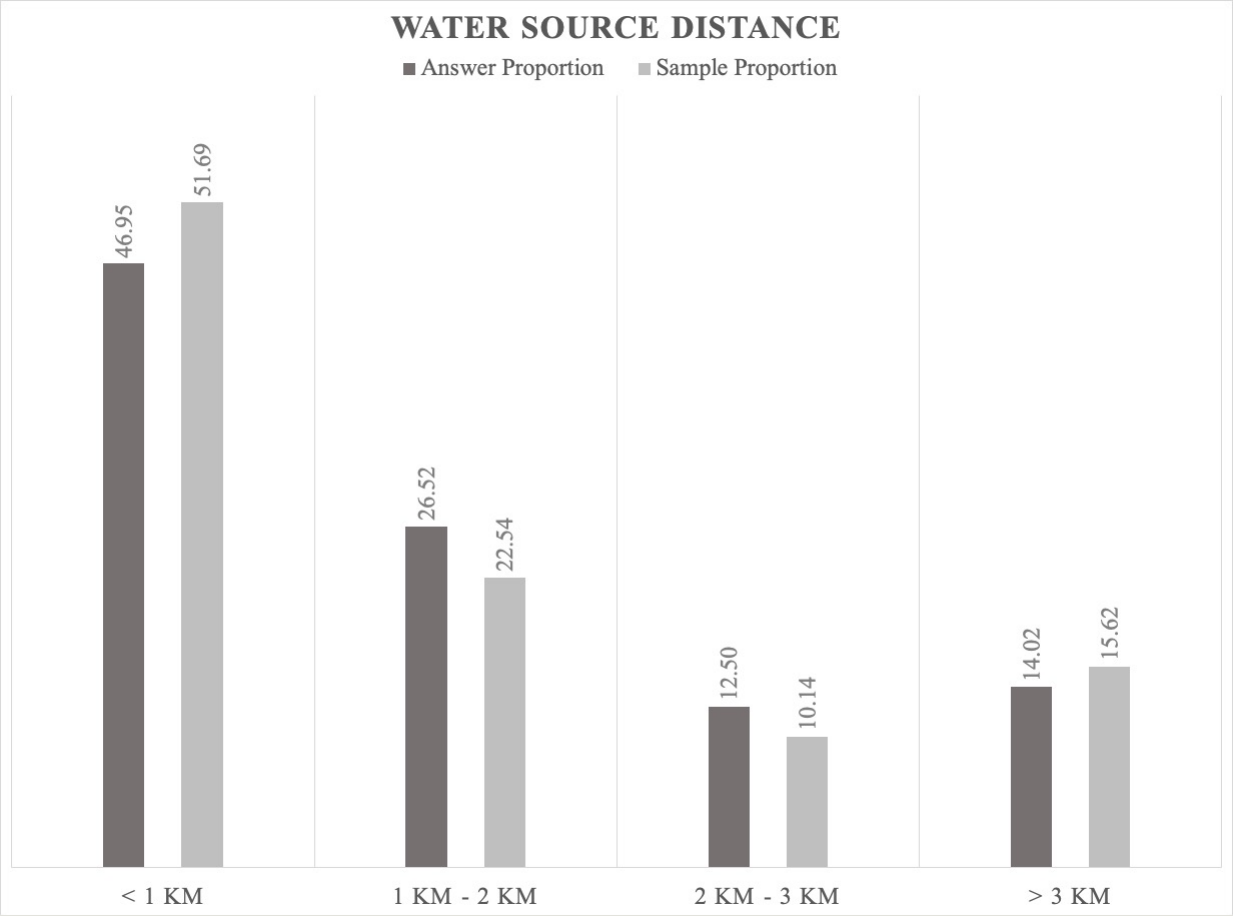
Proportion relationship between the jackals' positive answer and the sample regarding the water source as a factor that influences the jackal distribution.

1. Regarding *the altitude*, the largest answer proportion was located in areas with an altitude less than 500 m a.s.l. (Fig. 6). In higher regions (> 500 m and > 1000m), despite the sample proportion, the positive answers were low. This confirms that jackals prefer lowlands with moderate slopes and avoid mountainous regions with steep terrain, deep snow and covered by dense forest vegetation (see also point 2; Fig. 7).

2. Concerning *the vegetation type*, we found that the highest proportion of jackals' positive answers were in agricultural lands (to some degree, this result is also a suggestion for human-dependence in environmental preferences) and mixed landscape between open areas and scrublands (Fig. 8). In woodlands with a significant sample proportion, we found a low response.

3. Regarding *the wolf distribution* in Bulgaria, the habitats for the reproduction of the wolf are generally not inhabited by jackals. However, the limiting factor in this case is related not only to the wolf's presence but also to the unfavourable landscape for jackals as steep terrain, dense forestand deep snow (see point 1). The data showed positive answers of jackals in wolf reproductive territory (after the map of wolf distribution in Bulgaria, see: Spiridonov and Spassov 2015) (Fig. 9). As we mentioned before, wolves and jackals can co-exist when food and hiding places are sufficiently abundant.

4. Additional factors with probable influence on jackal distribution (important also for wolves): we found that there were more positive answers in areas near water sources (< 1 km). However, it is important to note that, in this case, the positive answers were directly proportional to the sample size (Fig. 10), so this difference could also be influenced by the sample effort.

**Factors determining the expansion of jackal’s population in Europe.** The distribution of the species from the Balkans to Pribaltic Region for the last 30 years and the significant enhancement of its population on the Balkans (over 30,000 jackals registered in Bulgaria only: Stoyanov 2013), demonstrate the recent expansion of its population. Several factors have been mentioned in literature as an explanation for this phenomenon: land use changes (Šálek et al. 2013) and climate change (Giannatos 2004, Arnold et al. 2011, Musabekov et al. 2016, Pyšková et al. 2016). Furthermore, the species expansion may be easier where wolves, natural intra-guild predators of golden jackals, are uncommon or absent (Trouwborst et al. 2015; but see above).

**Figure 11.**
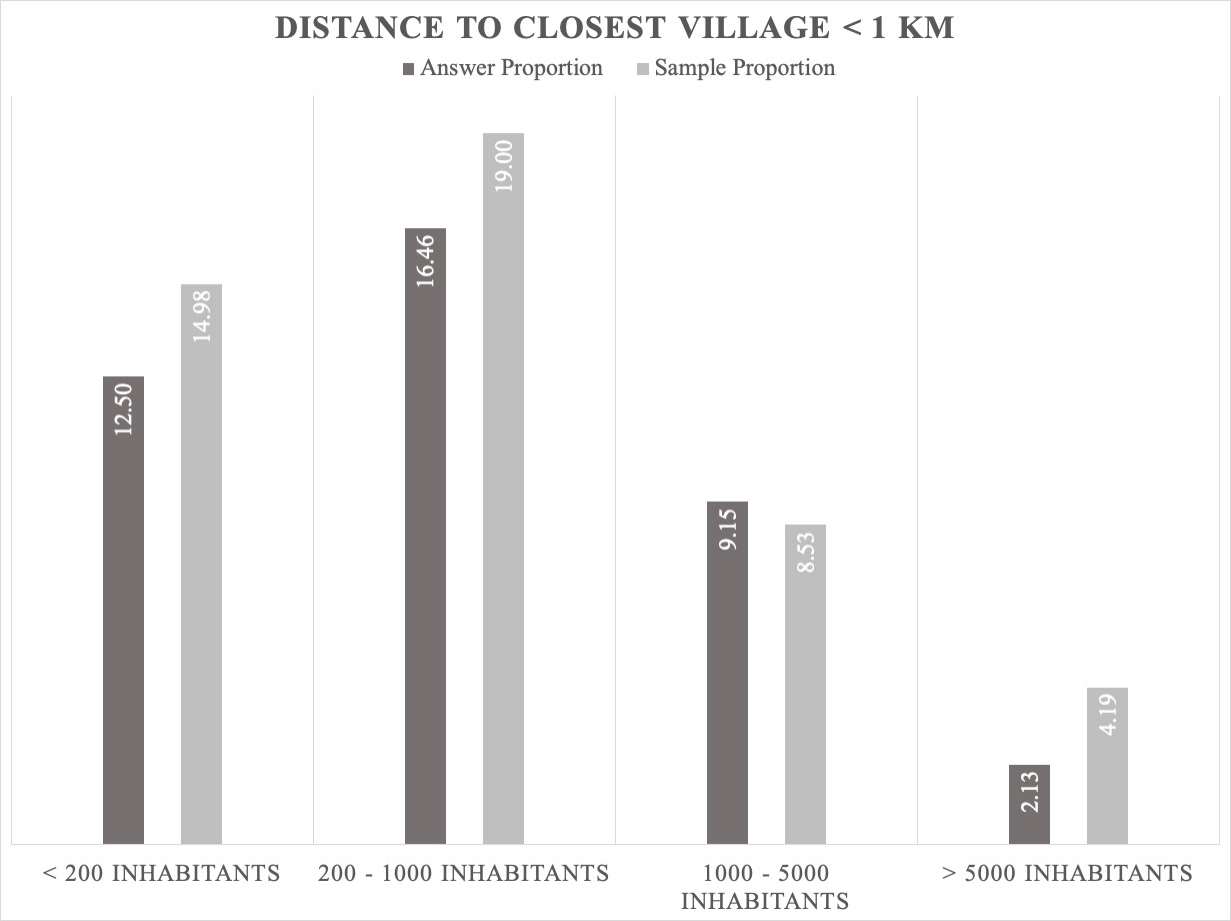
Proportion relationship between the jackals' positive answer and the sample regarding human settlements as a factor that influences the jackal distribution (population density; distance from the calling station < 1 km).

**Figure 12.**
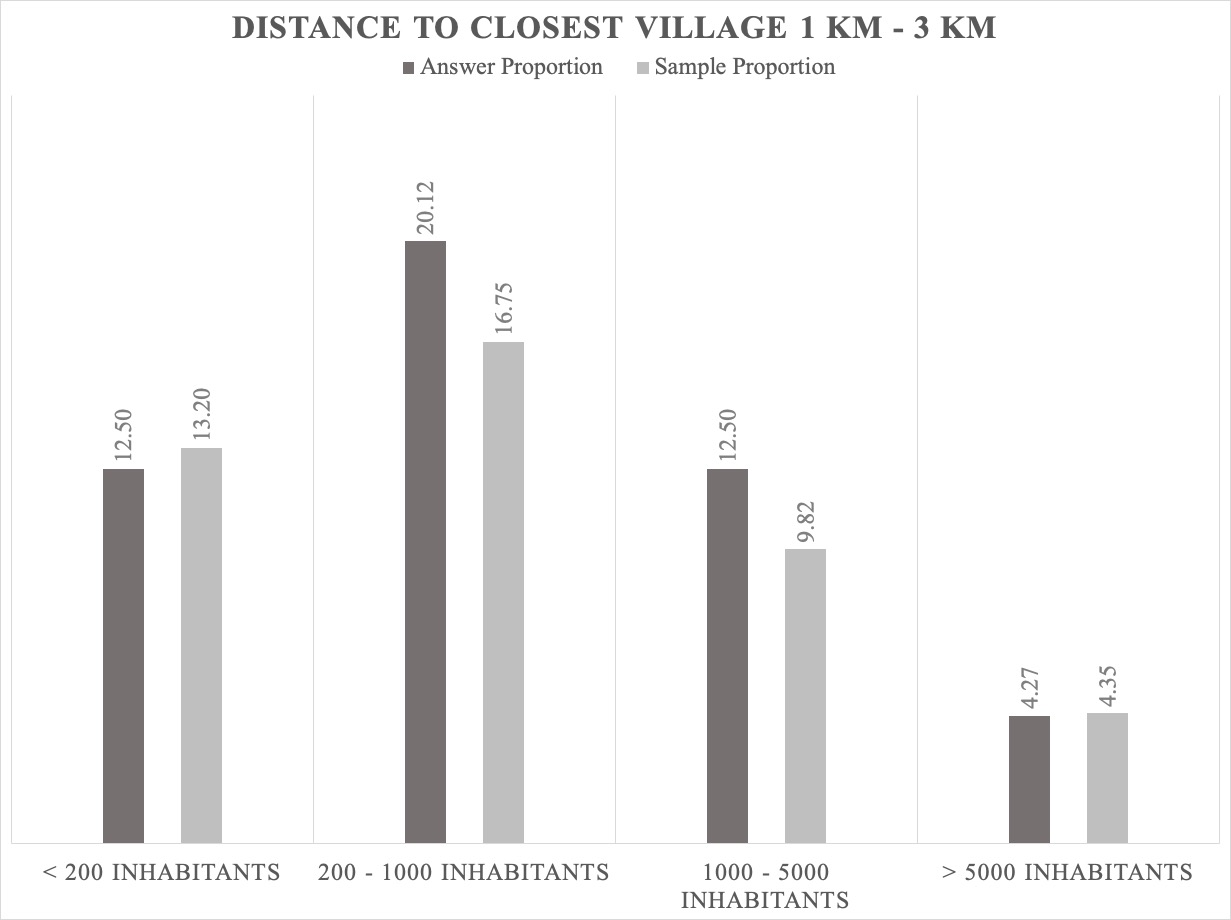
Proportion relationship between the jackals' positive answer and the sample regarding human settlements as a factor that influences the jackal distribution (population density; distance from the calling station between 1 and 3 km).

**Figure 13.**
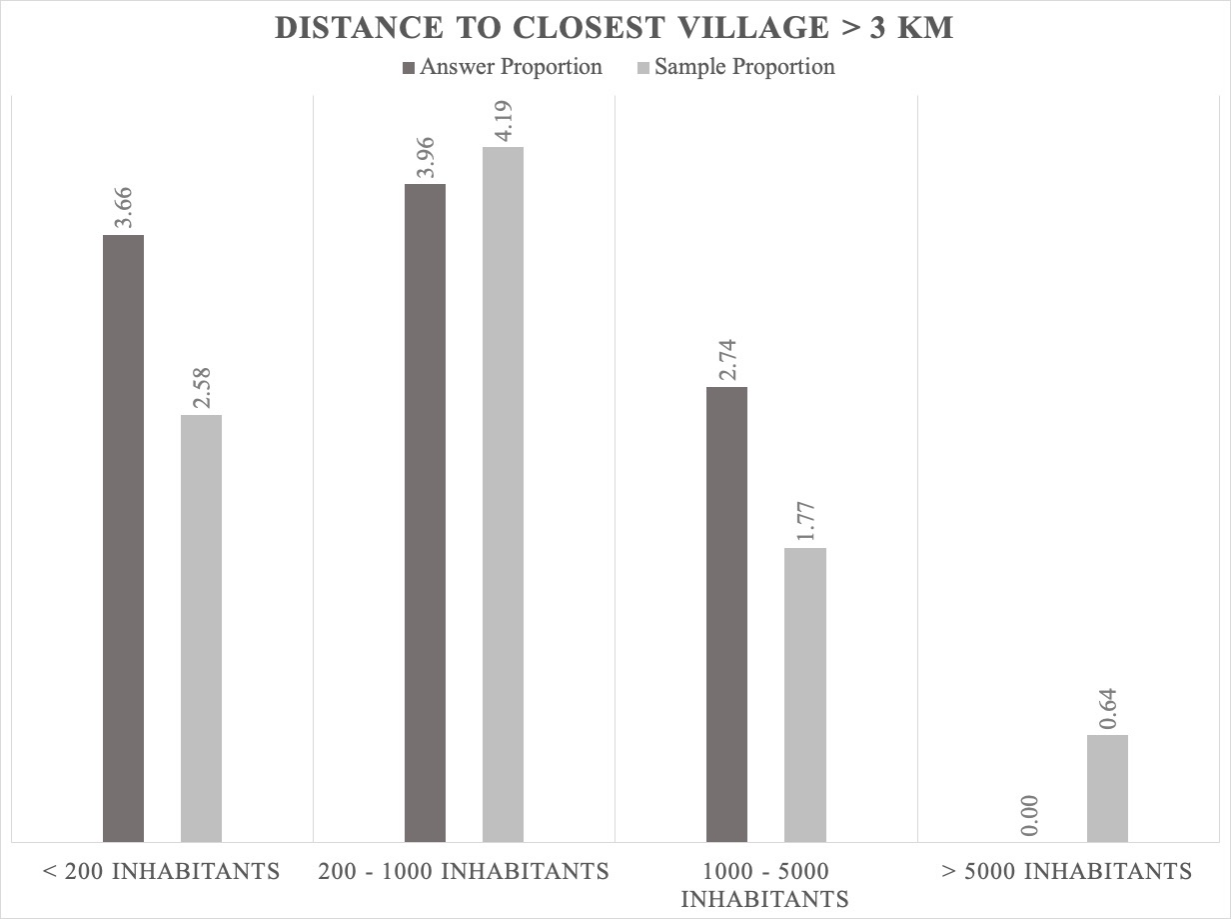
Proportion relationship between the jackals' positive answer and the sample regarding human settlements as a factor that influences the jackal distribution (population density; distance from the calling station > 3 km).

**Figure 14.**
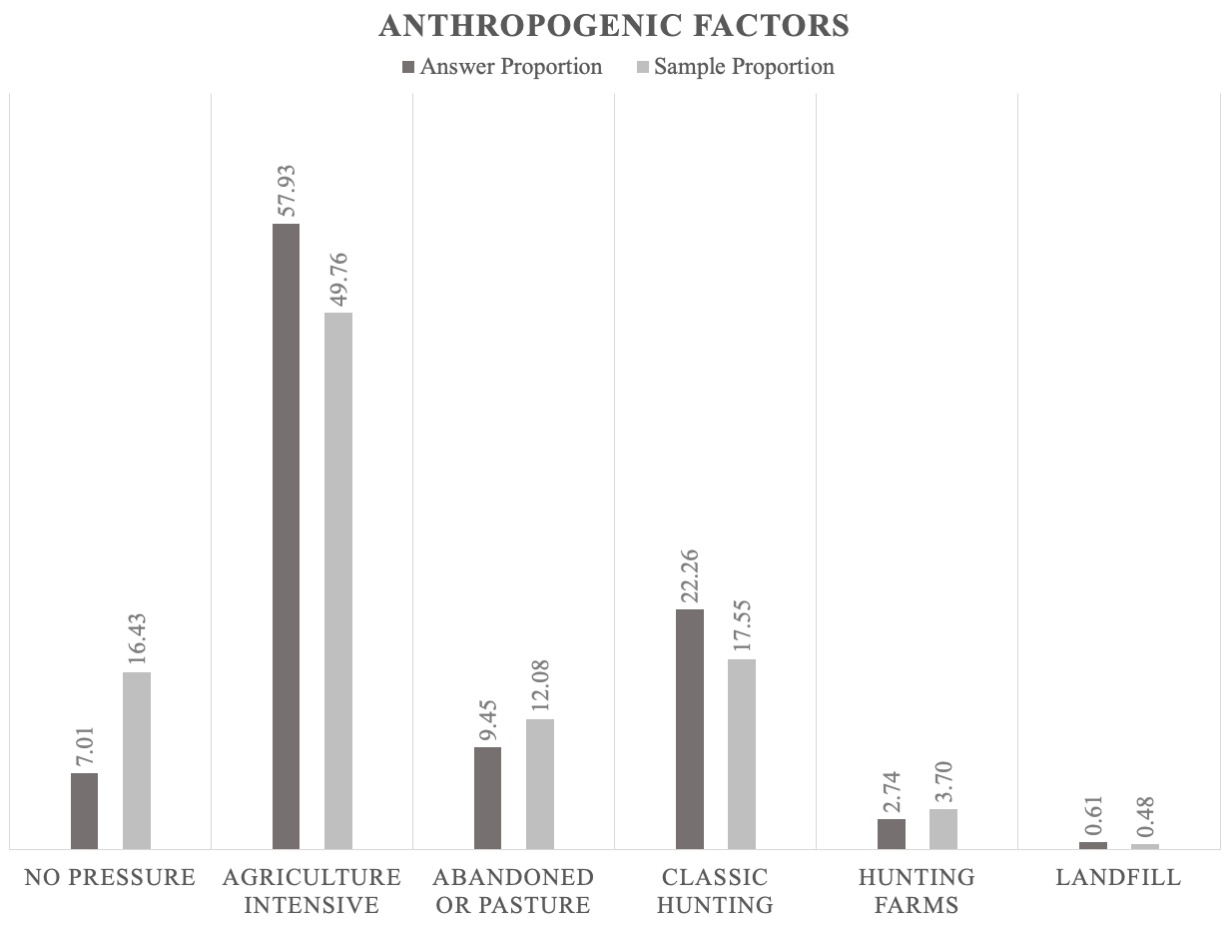
Proportion relationship between the jackals' positive answer and the sample regarding the anthropogenic activity as a factor that influences the jackal distribution.

A unique combination of factors (mentioned below), caused by human activity in Europe, could also be responsible for the population explosion:

a. Deforestation;

b. Development of a network of roads;

c. Additional food sources, related to human activities: settlements with villages that producelarge amounts of food waste and agricultural activities;

The jackals prefer to be close to human settlements where there are scavenging opportunities (Giannatos 2004, Giannatos et al. 2005). However, the data gathered by our bioacoustic monitoring show that the jackals' positive answer proportion is not dependent on the distance to human settlements; the results indicate that the answers are directly proportional to the sample (Figs 11, 12, 13). Jackals' response suggests that this species avoids places with higher human population densities (over 1000 inhabitants), since the largest answer proportion has been registered in areas close to villages that have between 200 and 1000 inhabitants (Figs 11, 12, 13). The intensive agriculture offers a favourable environment for the jackal, the percentages between answer and sample being directly proportional. In areas without anthropogenic factors, the answer proportion is low, despite the sample effort (Fig. 14). This gives certain indications that the jackal does not prefer these types of habitats and that it is more common in open spaces with agriculture and mixed landscapes (Fig. 8).

d. Decrease of the wolf population (see the discussion above);

e. On the other hand, the simultaneous growth of the Balkan and Caucasian populations indicates that the expansion of those two distant populations could be a result of potential common factors such as climate changes and global warming. However, this hypothesis mostly applies to the recent penetration of the species into the most northern territories and is less applicable to the initial phase of population explosion, for example on the Balkans, which has happened in mild climatic conditions. The common reason for expansion should be related to the similar impact of the anthropogenic influence and the combination of the above-cited factors.

In addition, it should be considered that the jackal is extremely adaptive (Šálek et al. 2013, Pyšková et al. 2016) and more adapted to exist in landscapes, modified by man as compared to the wolf (Krofel et al. 2017).

To synthesise, the exact chronology of the penetration of *Canis aureus moreoticus* in Europe remains unclear. There are no fossils, neither subfossil remains from the species even in the south-eastern parts of the continent, which suggests the possibility for a later dispersal, potentially related to anthropogenic activities. Historical data show that the typical habitats of the jackals are in South-Eastern Europe with some penetration areas in the north and west during certain favourable periods. The current expansion to the continent represents the largest population explosion of the species within these territories. It has started from only three basal population nuclei: from the Balkans (the Peri-Strandja area and the Dalmatian coast) and Caucasus (initially from the east parts of Western Transcaucasia; see Fig. 5). This expansion to the west and north has been the result of the unique combination of factors with anthropogenic origin.

## Supplementary Material

XML Treatment for Canis aureus moreoticus
